# HOPS-Dependent Endosomal Escape Demands Protein Unfolding

**DOI:** 10.1021/acscentsci.4c00016

**Published:** 2024-03-26

**Authors:** Madeline Zoltek, Angel L. Vázquez Maldonado, Xizi Zhang, Neville Dadina, Lauren Lesiak, Alanna Schepartz

**Affiliations:** † Department of Molecular and Cell Biology, University of California, Berkeley, California 94720, United States; ‡ Department of Chemistry, University of California, Berkeley, California 94720, United States; § California Institute for Quantitative Biosciences, University of California, Berkeley, California 94720, United States; ∥ Chan Zuckerberg Biohub, San Francisco, California 94158, United States

## Abstract

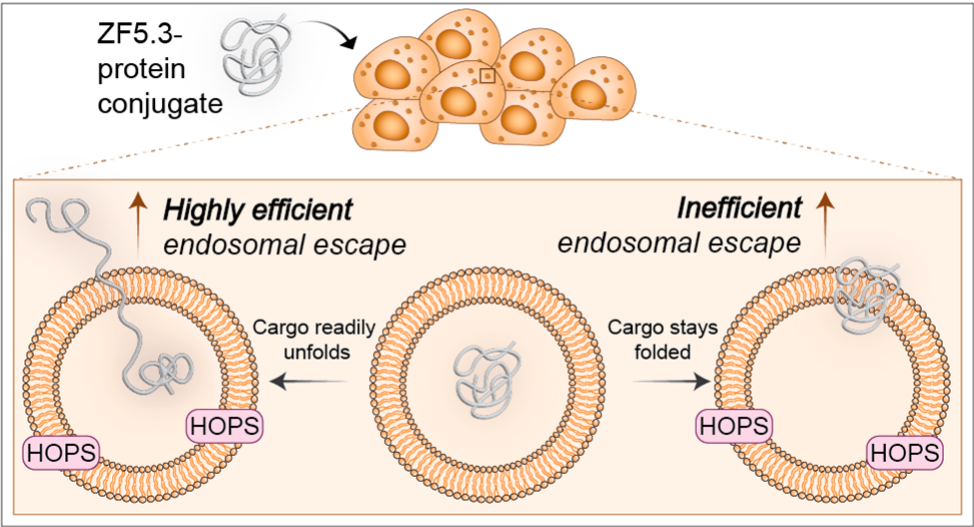

The inefficient translocation of proteins across biological membranes limits their application as potential therapeutics and research tools. In many cases, the translocation of a protein involves two discrete steps: uptake into the endocytic pathway and endosomal escape. Certain charged or amphiphilic molecules can achieve high protein uptake, but few are capable of efficient endosomal escape. One exception to this rule is ZF5.3, a mini-protein that exploits elements of the natural endosomal maturation machinery to translocate across endosomal membranes. Although some ZF5.3–protein conjugates are delivered efficiently to the cytosol or nucleus, overall delivery efficiency varies widely for different cargoes with no obvious design rules. Here we show that delivery efficiency depends on the ability of the cargo to unfold. Using fluorescence correlation spectroscopy, a single-molecule technique that precisely measures intracytosolic protein concentration, we show that regardless of size and pI, low-*T*_m_ cargoes of ZF5.3 (including intrinsically disordered domains) bias endosomal escape toward a high-efficiency pathway that requires the homotypic fusion and protein sorting (HOPS) complex. Small protein domains are delivered with moderate efficiency through the same HOPS portal, even if the *T*_m_ is high. These findings imply a novel pathway out of endosomes that is exploited by ZF5.3 and provide clear guidance for the selection or design of optimally deliverable therapeutic cargo.

## Abstract 2

The cell-permeant mini-protein ZF5.3 crosses endosomal membranes most efficiently to deliver protein cargoes to the cell cytosol when the cargo is capable of unfolding under physiological conditions.

## Introduction

Protein- and nucleic-acid-derived biologics are a rapidly expanding sector of modern drug development. When compared to small molecules, biologics can improve target specificity, inhibit or activate recalcitrant targets, replace missing or malfunctioning enzymes, and deliver gene editing or protein-editing machineries.^1^ Direct protein delivery is simpler than lipid nanoparticle or viral vector delivery strategies^2^ and provides fine-tuned control over dosage and intracellular lifetime. Despite this potential, there is not a single approved protein therapeutic that operates in the cytosol or nucleus. The problem is poor endosomal escape. Decades of research dedicated to improving the endosomal escape of proteins delivered via the endosomal pathway have yielded many molecules that stimulate endocytic uptake, but almost none that escape endosomes and avoid a degradative fate.

One molecule that has shown promise with regard to endosomal escape is ZF5.3, a 27-aa mini-protein that exploits the HOPS complex, a natural and ubiquitous component of the endosomal maturation machinery,^3−6^ to guide certain proteins into the cytosol and nucleus.^6−9^ A conjugate of ZF5.3 and the transcription factor MeCP2 (implicated in Rett Syndrome) reaches the nucleus of mammalian cells with an efficiency of >80% (defined as nuclear concentration divided by treatment concentration) while retaining its native binding partners and function.^6^ The delivery of ZF5.3–MeCP2 is substantially more efficient than that of other ZF5.3–protein conjugates^7,8^ and to our knowledge any other reported nucleic acid or protein biologic that escapes the endocytic pathway. Precisely which attributes of ZF5.3–MeCP2 enable such efficient endosomal escape, and whether these attributes could be generalized, however, remain unclear.

Endosomal escape of a biologic requires the energetically unfavorable translocation of a hydrophilic molecule across a hydrophobic membrane. Nature overcomes the challenges of protein translocation in many cases through two distinct mechanisms. One mechanism requires unfolding of the protein being transported (e.g., via Sec-translocases^11,12^ or mitochondrial import pathways^13−15^), whereas the other accommodates the globular fold of the protein in transit (e.g., during peroxisome entry^16^ or unconventional protein secretion^17,18^). Regardless of the cellular machinery required, given that the structure of MeCP2 is up to 60% disordered,^6,19^ we hypothesized that intrinsic disorder could favor endosomal escape through a pathway that demands protein unfolding.

Here we test this hypothesis and discover that the ability to unfold is a key determinant in how well ZF5.3 guides a protein into the cytosol in a HOPS-dependent manner. Proteins that are intrinsically disordered or unfold at physiological temperatures are delivered into the cytosol by ZF5.3 with high efficiency and in a HOPS-dependent manner. We also discovered that proteins with greater thermal stability can be delivered with modest efficiency and in a HOPS-dependent manner if the domain is sufficiently compact. Super-resolution microscopy images of endolysosomes in ZF5.3-treated cells provide evidence for distinct condensed subpopulations that associate with the limiting membrane. Our data support a model in which intrinsically disordered proteins or those that unfold readily are privileged with respect to efficient endosomal escape via a HOPS-dependent portal. We anticipate that these design rules will constitute a useful filter in the development of direct protein delivery strategies and provide new insights into how proteins, natural or designed, circumnavigate biological membranes.

## Results

To establish whether unfolding plays a role in ZF5.3-mediated endosomal escape, we built on classic work of Eilers and Schatz, who almost 40 years ago utilized the ligand-dependent stability of the enzyme dihydrofolate reductase (DHFR) to study protein import into mitochondria.^13^ The thermal stability of DHFR (*T*_m_) increases by approximately 15 °C upon the binding of ligands such as methotrexate (MTX) or trimethoprim.^13^ Indeed, the effect of MTX or trimethoprim on protein import and export established a role for protein unfolding during chaperone-mediated lysosomal import mediated by heat shock family molecular chaperones,^20,21^ protein translocation across the *E. coli* plasma membrane mediated by the Sec-translocase,^11,12^ endoplasmic reticulum retrotranslocation,^22^ and cytosolic delivery of toxins such as ricin and diphtheria.^23−25^

**Figure 1:**
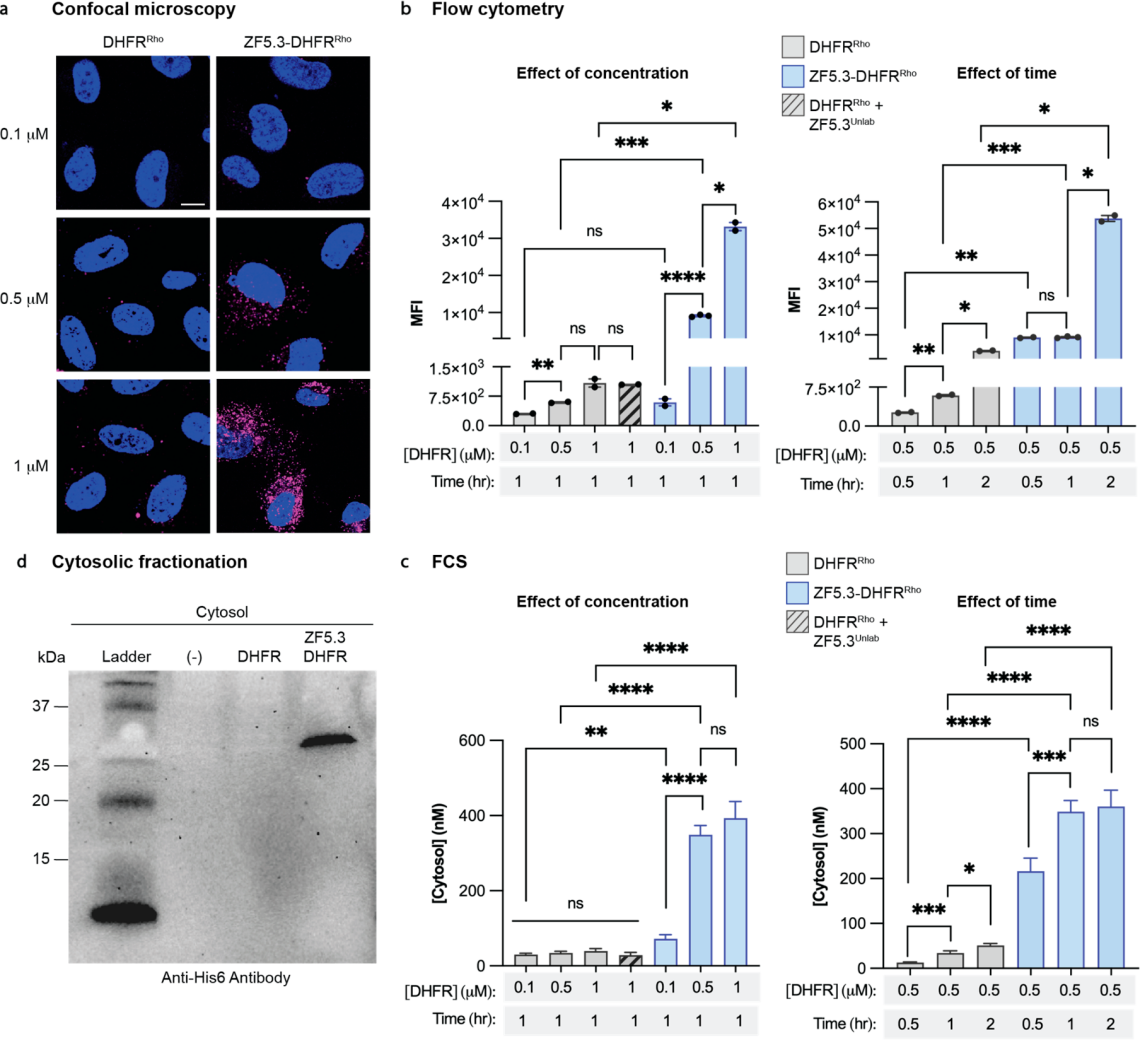
DHFR reaches the cytosol efficiently when fused covalently to ZF5.3. (A) 2D confocal microscopy images of Saos-2 cells incubated with the indicated concentration of DHFR^Rho^ or ZF5.3–DHFR^Rho^ as described in Supporting Information (SI) Methods. Scale bar: 10 μm. Plots showing (B) flow cytometry (FC) analysis of total cellular uptake or (C) fluorescence correlation spectroscopy (FCS) analysis of cytosolic concentrations of DHFR^Rho^, ZF5.3–DHFR^Rho^, or a 1:1 mixture of ZF5.3 and DHFR^Rho^ after the indicated treatment concentration and incubation time; see SI Methods for the detailed procedure. FC values are provided as median fluorescence intensity (MFI) for the lissamine rhodamine B channel; *n* = 20 000 in total per condition containing at least two biological replicates each (mean ± SEM). FCS values provided in nM; *n* > 20 for each FCS condition with two biological replicates each (mean ± SEM). Statistical significance comparing the given concentrations was assessed using the Brown–Forsythe and Welch one-way analysis of variance (ANOVA) followed by an unpaired *t* test with Welch’s correction. ****p ≤ 0.0001, ***p ≤ 0.001, **p ≤ 0.01, *p ≤ 0.05. (D) Western blot analysis of fractionated cytosol from Saos-2 cells treated with either DMEM media alone (−), DHFR, or ZF5.3–DHFR at 1 μM for 1 h. The presence of intact DHFR or ZF5.3–DHFR was assessed by using an anti-His6 antibody. The gel results shown are representative of two biological replicates.

We purified samples of DHFR and ZF5.3–DHFR from *E. coli* and confirmed their identities using SDS-PAGE and LC/MS (Figures S1a–S1c). The presence of ZF5.3 at the N-terminus of DHFR has little or no effect on overall protein secondary structure or catalytic activity (Figures S1d and S1e). With these materials in hand, we established baseline values for the cytosolic delivery of DHFR and ZF5.3–DHFR using rhodamine-tagged variants (DHFR^Rho^ and ZF5.3–DHFR^Rho^) prepared using sortase, as described previously (Figures S1a–S1c).^6−8^ We incubated human osteosarcoma (Saos-2) cells with 0.1–1 μM DHFR^Rho^ or ZF5.3–DHFR^Rho^ for 1 h, washed and trypsin-treated the cells to remove surface-bound material, and visualized the cells using confocal microscopy, flow cytometry (FC), and fluorescence correlation spectroscopy (FCS) (Figure 1 and Figures S2 and S3). Confocal microscopy and FC revealed that cells treated with ZF5.3–DHFR^Rho^ showed a substantially higher total intracellular fluorescence than those treated with DHFR^Rho^ at all treatment concentrations and time points. The overall uptake of DHFR^Rho^ and ZF5.3–DHFR^Rho^ revealed by confocal microscopy (Figure 1a and Figure S2) and FC (Figure 1b) was dose-dependent; the total uptake of ZF5.3–DHFR^Rho^ was significantly higher than that of DHFR^Rho^, especially at treatment concentrations of 0.5 μM (15.5-fold increase) and 1 μM (30.6-fold increase). These increases in total uptake due to fusion to ZF5.3 are in line with values measured for other ZF5.3–protein conjugates.^7,8^ No increase in uptake was observed when cells were treated with a 1:1 mixture of ZF5.3 and DHFR^Rho^ (Figure 1b), confirming that improved uptake demands a covalent linkage to the cargo.^8^

Although endocytic uptake is the first step along the pathway to the cytosol, the key determinant of delivery efficiency is endosomal escape, or the fractional concentration of intact protein that reaches the cytosol. Two challenges have thwarted attempts to improve cytosolic delivery. The first is the absence of tools to accurately quantify how much material actually reaches the cytosol (delivery efficiency), and the second is the difficulty in establishing whether the delivered material is intact (or not) and thus capable of function. We used live cell FCS^26^ to establish delivery efficiency^27^ by quantifying the concentration of DHFR^Rho^ and ZF5.3–DHFR^Rho^ that reached the cytosol of Saos-2 cells. Unlike flow cytometry, FCS provides both the concentration and the diffusion time of a fluorescent molecule within a subcellular compartment, such as the cytosol or nucleus.^27−30^ The former value provides an accurate measure of delivery efficiency, while the latter, when combined with careful biochemistry, establishes whether the fluorescent material is intact.^27,31^

### ZF5.3–DHFR^Rho^ Trafficks Efficiently into the Saos-2 Cytosol

Examination of treated Saos-2 cells using FCS revealed substantial differences in the efficiencies with which DHFR^Rho^ and ZF5.3–DHFR^Rho^ reached the cytosol. Cells treated with DHFR^Rho^ showed little trafficking of this material to the cytosol at any concentration studied (Figure 1c and Figure S3). At the highest treatment concentration (1 μM), the measured cytosolic concentration of DHFR^Rho^ was 39 nM, with a delivery efficiency of only 3.9%. By contrast, ZF5.3–DHFR^Rho^ reached the cytosol efficiently and in a dose-dependent manner, establishing average concentrations of 72, 350, and 393 nM when cells were treated with 0.1, 0.5, and 1 μM ZF5.3–DHFR^Rho^, respectively, for 1 h (Figure 1c). These values correspond to delivery efficiencies between 39% and 72%, up to 10-fold higher than those measured for DHFR^Rho^. Notably, at a fixed treatment concentration of 0.5 μM ZF5.3–DHFR^Rho^, additional incubation time (up to 2 h) improves total uptake but does not substantially increase the fraction that reaches the cytosol (Figures 1b and 1c). These data suggest that ZF5.3–DHFR^Rho^ follows a saturable pathway to escape from endosomes and that endosomal escape (as opposed to an earlier endocytic event) kinetically limits delivery to the cytosol. When stringently isolated from the cytosol of treated cells, ZF5.3–DHFR was recovered fully intact with no evidence of either degradation or endosomal contamination (Figure 1d and Figure S4). Co-administration of ZF5.3 did not improve the cytosolic delivery of DHFR^Rho^, confirming that efficient delivery demands a covalent linkage of ZF5.3 to the cargo^8^ (Figure 1c). Thus, the presence of ZF5.3 at the N-terminus of DHFR^Rho^ improved its delivery to the cytosol by up to 10-fold. The cytosolic delivery of ZF5.3–DHFR^Rho^ is more efficient than nearly all other proteins delivered by ZF5.3 previously,^7,8^ and though it is not intrinsically disordered, the translocation efficiency of ZF5.3–DHFR^Rho^ into the cytosol mirrors that of ZF5.3–MeCP2.^6^

### Delivery of DHFR by ZF5.3 Is Inhibited by Equimolar MTX

**Figure 2:**
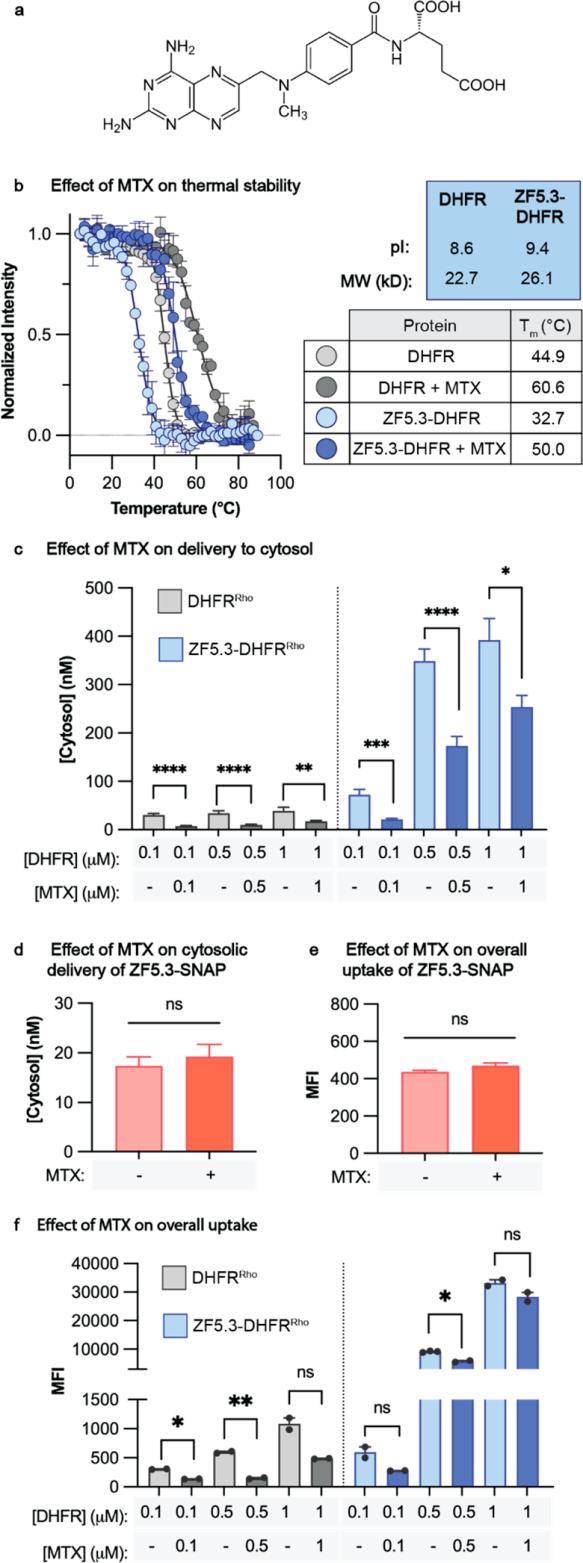
Delivery of DHFR by ZF5.3 is inhibited by equimolar MTX. (A) Chemical structure of methotrexate (MTX). (B) Plots illustrating the temperature-dependent loss in the circular dichroism (CD) signal at 210 nm for DHFR and ZF5.3–DHFR (20 μM protein in 25 mM Tris, 150 mM KCl, 1 mM TCEP, pH 7.2) in the presence or absence of 1 equiv of MTX. For each melt, the temperature was increased in 2° increments between 5 and 90 °C, and the ellipticity at 210 nm was fitted using a Boltzmann sigmoidal nonlinear regression. The melts were irreversible, and therefore we report the midpoint value of these fitted curves as apparent *T*_m_ values (*T_m_). The data shown include two biological replicates. (C–F) Plots illustrating the effect of MTX on the cytosolic delivery (C–D) and overall uptake (E–F) of DHFR^Rho^, ZF5.3–DHFR^Rho^, or ZF5.3–SNAP^Rho^. In all cases, the proteins were preincubated with 1 equiv of MTX for 30 min and then added to cells at a total treatment concentration of 0.1 – 1 μM for 1 h (DHFR^Rho^ and ZF5.3–DHFR^Rho^) or 1 μM for 30 min (ZF5.3–SNAP^Rho^). Cells were then trypsinized and analyzed by FC or FCS as described in SI Methods. FC values are provided as median fluorescence intensity for the lissamine rhodamine B channel (MFI); *n* = 20 000 per condition in total with at least two biological replicates each (mean ± SEM). FCS values are provided in nM; *n* > 20 for each FCS condition comprising two biological replicates each (mean ± SEM). Statistical significance comparing the given concentrations was assessed using the Brown–Forsythe and Welch one-way analysis of variance (ANOVA) followed by an unpaired *t* test with Welch’s correction. ****p ≤ 0.0001, ***p ≤ 0.001, **p ≤ 0.01, *p ≤ 0.05.

Next, to interrogate the role of protein folding in cytosolic delivery mediated by ZF5.3, we determined the impact of the DHFR-selective inhibitor methotrexate (MTX, Figure 2a) on the cytosolic delivery efficiencies of DHFR^Rho^ and ZF5.3–DHFR^Rho^. MTX binds DHFR with subnanomolar affinity (*K*_D_ ≈ 10^–10^ M)^32^ and potently inhibits enzyme activity^33^ (Figure S1e). Temperature-dependent circular dichroism (CD) spectroscopy established that the apparent thermal stabilities (*T_m_) of DHFR and ZF5.3–DHFR increased by approximately 15 degrees in the presence of 1 equiv MTX. For DHFR, the *T_m_ measured in the absence of MTX was 44.5 °C, in line with previous measurements,^34^ and increased by 16.6 °C in the presence of 1 equiv MTX. For ZF5.3–DHFR, the *T_m_ in the absence of MTX was 32.7 °C and the corresponding increase was 17.3 °C (Figure 2b).

Samples of DHFR^Rho^ and ZF5.3–DHFR^Rho^ at concentrations from 0.1–1 μM were preincubated with 1 equiv MTX for 30 min, added to Saos-2 cells, and incubated for 1 h as described previously. Under all conditions, the presence of 1 equiv of MTX substantially decreased the fraction of ZF5.3–DHFR^Rho^ that reached the cytosol (Figure 2c). The effect of MTX was inversely related to the ZF5.3–DHFR^Rho^ concentration, with reductions of 70.4%, 50.4%, and 42.8% at incubation concentrations of 0.1, 0.5, and 1 μM, respectively (Figure 2c). Notably, MTX also decreased the concentration of DHFR^Rho^ that reached the cytosol by comparable amounts, but had no effect on the cytosolic delivery of ZF5.3–SNAP^Rho^, a protein that is not known to interact substantially with MTX (Figure 2d).

To evaluate the extent to which MTX affected cytosolic delivery by inhibiting the overall uptake of ZF5.3–DHFR^Rho^, we also evaluated treated cells using flow cytometry (Figure 2e). These results indicate that MTX has different effects on the overall uptake of DHFR^Rho^ and ZF5.3–DHFR^Rho^. Although one equivalent of MTX substantially decreased the overall uptake of DHFR^Rho^ by between 55% and 75% at all treatment concentrations, there was little or no effect of MTX on the overall uptake of ZF5.3–DHFR^Rho^ at treatment concentrations of 0.5 and 1 μM. MTX had no effect on the overall uptake of the unrelated protein ZF5.3–SNAP^Rho^ (Figure 2e). The observation that MTX has a substantial effect on delivery of ZF5.3–DHFR^Rho^ to the cytosol but little or no effect on overall uptake implies that unfolding plays a significant role in one or more of the steps that guides ZF5.3–DHFR^Rho^ out of the endocytic pathway and into the cytosol. For this reason, the relatively low thermostability (*T*_m_ = 32.7 °C) of ZF5.3–DHFR likely contributes to its highly efficient endosomal escape. These data also suggest that the endosomal uptake and escape of DHFR^Rho^ and ZF5.3–DHFR^Rho^ proceed using fundamentally different molecular machinery or pathways, but only the pathway accessed by ZF5.3–DHFR results in efficient cytosolic delivery.

### Unfolding of Cargo Is a General Requirement for High-Efficiency ZF5.3 Delivery

**Figure 3:**
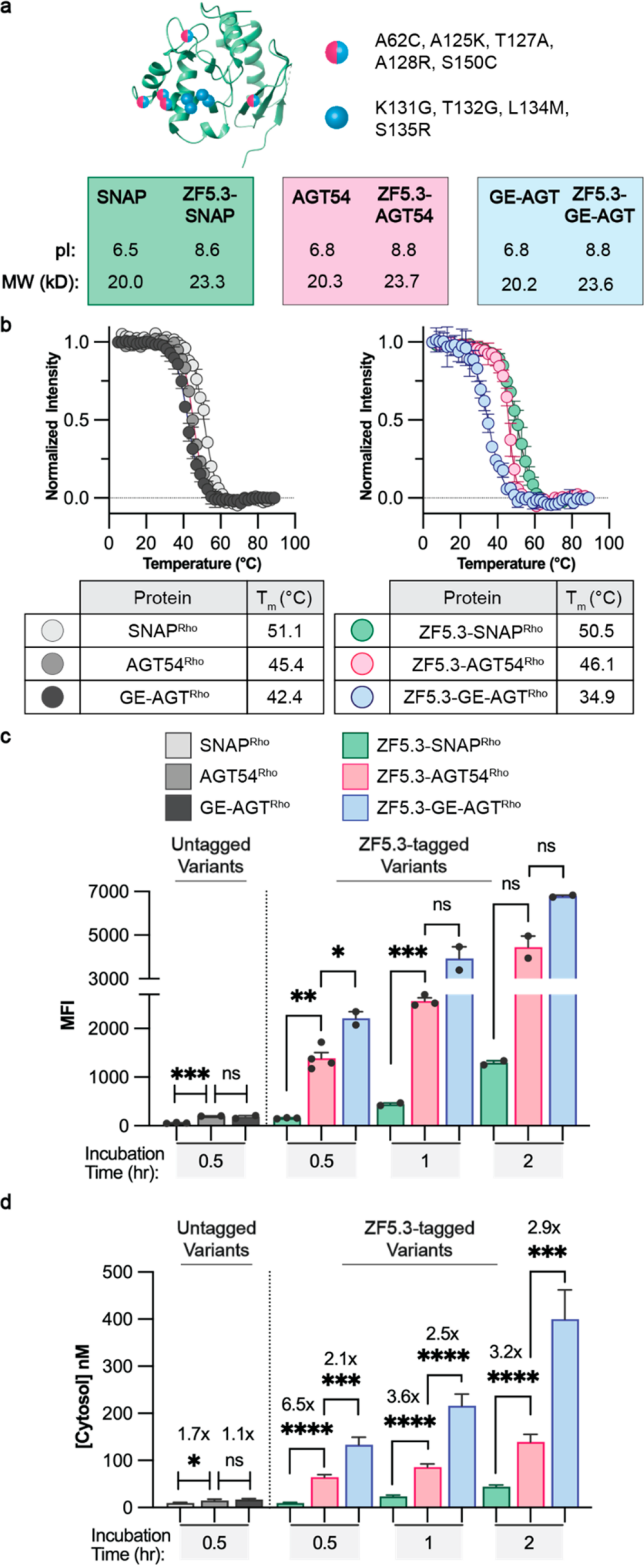
Lowering the thermal stability of SNAP-tag improves delivery to the cytosol only upon conjugation to ZF5.3. (A) Structure of SNAP-tag (PDB: 6Y8P) with destabilizing mutations marked by magenta and blue circles (for residues found in both AGT54 and GE-AGT) or blue circles (for residues found only in GE-AGT). Both the protein isoelectric point (pI) and molecular weight (in kilodaltons, kDa) increase upon the addition of ZF5.3 but remain comparable among all variants. (B) Plots illustrating the temperature dependence of the 222 nm CD signal of 20 μM GE-AGT^Rho^, AGT54^Rho^, and SNAP^Rho^ alongside the corresponding ZF5.3 conjugates in 20 mM Tris and 150 mM NaCl, pH 7.5. For each melt, the temperature was increased in 2° increments between 5 and 90 °C and the ellipticity at 222 nm was fitted using a Boltzmann sigmoidal nonlinear regression to obtain the *T_m_ values. The data shown include two biological replicates. (C–D) Total cellular uptake (top) and cytosolic concentration (bottom) of the untagged or ZF5.3-tagged SNAP^Rho^ variants as determined using FC and FCS, respectively. Saos-2 cells were incubated with 1 μM of the indicated protein for 30 min, 1 h, or 2 h before cellular workup, and measurements were performed as described previously. Flow cytometry values are provided as median fluorescence intensity for the lissamine rhodamine B channel; *n* = 20 000 per condition in total with two biological replicates each (mean ± SEM). FCS values are provided in nM; *n* > 20 for each FCS condition with two biological replicates each (mean ± SEM). Statistical significance was assessed using the Brown–Forsythe and Welch one-way analysis of variance (ANOVA) followed by an unpaired *t* test with Welch’s correction. ****p ≤ 0.0001, ***p ≤ 0.001, **p ≤ 0.01, *p ≤ 0.05.

Although one equivalent of MTX inhibits the fraction of ZF5.3–DHFR^Rho^ that reaches the cytosol (Figure 2c), the inhibition is partial, not complete. We reasoned that this finding might be due to the loss of MTX from ZF5.3–DHFR^Rho^ before the complex reaches the endosomal compartment from which escape occurs, especially as the compartments become progressively more acidic. To more directly evaluate the role of unfolding in endosomal escape, we turned to three known SNAP-tag variants that differ by only a few amino acid substitutions but nonetheless show distinctly different thermal stabilities.^35−38^ These variants, all intermediates generated along the directed evolution pathway between human O^6^-alkylguanine-DNA alkyltransferase and commercially available SNAP-tag, display thermal stabilities between 35–51 °C but with nearly indistinguishable molecular weights and isoelectric points of 8.7 ± 0.1 (Figure 3a and Figure S5a).^35^ Each SNAP-tag variant was conjugated to the C-terminus of ZF5.3 and tagged with rhodamine upon reaction with benzylguanine-modified lissamine rhodamine B (BG-Rho) (Figures S5b and S5c). Temperature-dependent CD studies confirmed the previously reported thermal stabilities; once again, the presence of ZF5.3 had a modest destabilizing effect on the *T_m_ but little or no effect on the overall secondary structure (Figure 3b and Figure S5d).

Saos-2 cells were treated with each SNAP^Rho^ variant (1 μM) for 0.5–2 h and evaluated using confocal microscopy, flow cytometry, and FCS as described previously (Figures 3c and 3d and Figures S6 and S7). The most stable variant (ZF5.3–SNAP^Rho^, *T_m_ = 51 °C) showed minimal uptake (Figure 3c) and poor trafficking to the cytosol (Figure 3d) regardless of incubation time, in line with results described previously for a closely related variant.^7^ The less thermostable proteins, ZF5.3–GE-AGT^Rho^ (*T_m_ = 35 °C) and ZF5.3–AGT54^Rho^ (*T_m_ = 46 °C), were taken up with higher efficiency but not equally when evaluated by flow cytometry, with uptake increasing after longer incubation times (Figure 3c). Given the roughly equal surface charges of SNAP, AGT54, and GE-AGT, it is interesting to note that decreased thermal stability seems to improve the overall ZF5.3-mediated cellular uptake.

Notably, the three ZF5.3–SNAP^Rho^ variants trafficked to the cytosol with different efficiencies, and in a manner that correlated directly with *T_m_ (Figure 3d and Figure S6). At all incubation times, ZF5.3–GE-AGT^Rho^, with the lowest *T_m_ (35 °C), reached the cytosol about 2.1–2.9-fold more efficiently than mid-*T_m_ ZF5.3–AGT54^Rho^, which in turn reached the cytosol 3.2–6.5-fold more efficiently than high-*T_m_ ZF5.3–SNAP^Rho^. At its maximum, the least thermostable variant ZF5.3–GE-AGT^Rho^ reached a concentration of 400 nM in the cytosol, corresponding to a 40% delivery efficiency; under equal conditions, ZF5.3–AGT54^Rho^ reached 139.2 nM and ZF5.3–SNAP^Rho^ only reached 44.2 nM. It is notable that ZF5.3–GE-AGT^Rho^ and ZF5.3–DHFR^Rho^ show comparable thermal stabilities (*T_m_ values of 35 and 33 °C, respectively) but ZF5.3–DHFR^Rho^ reaches the cytosol significantly more efficiently under comparable incubation conditions; this likely relates to the relatively higher total uptake of ZF5.3–DHFR^Rho^ (Figures 1b and 1c). On their own, the series of SNAP variants lacking ZF5.3 reached the cytosol at virtually undetectable levels (cytosolic concentrations between 9 and 16.8 nM after a 30 min incubation), with minimal differences among the three (Figures 3c and 3d), indicating that the relationship between thermostability and delivery is unique to a ZF5.3-driven pathway.

### ZF5.3-Mediated Delivery of a Small but Stable Monobody

**Figure 4:**
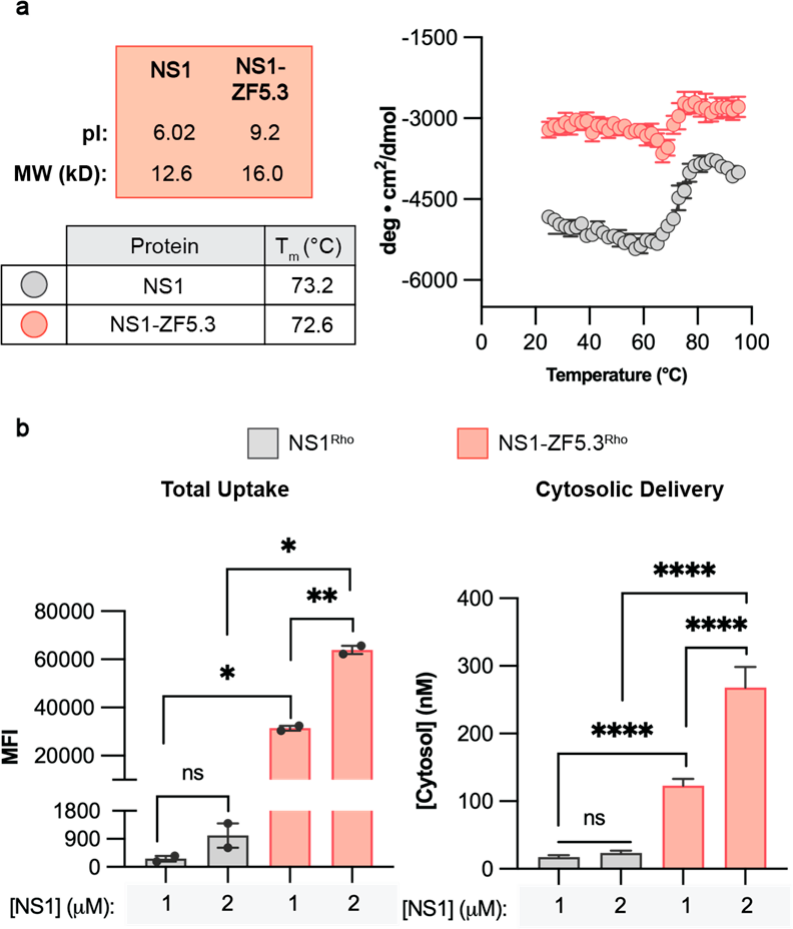
ZF5.3 can deliver the Ras-targeting monobody NS1 to the cytosol of cells. (A) Predicted isoelectric point (pI), molecular weight (kD), and experimentally determined *T_m_ (°C) of the Ras-targeting monobody NS1, with or without ZF5.3. The temperature-dependent CD signal at 218 nm was measured in 2° increments between 25 and 90 °C, and the ellipticity was fitted using a Boltzmann sigmoidal nonlinear regression to obtain the *T_m_ values. Each protein was measured in a buffer containing 20 mM Tris, 150 mM KCl, and 0.5 mM TCEP, pH 7.5. The observation that molar ellipticity decreases with temperature until ∼65 °C for both NS1 and NS1–ZF5.3 has been documented for fibronectin-like domains^48,49^ and may be due to a partial loss in structure at low temperatures. The (B) total cellular uptake (left) and cytosolic concentration (right) of NS1^Rho^ or NS1–ZF5.3^Rho^ were determined using FC and FCS, respectively. Saos-2 cells were incubated with 1 or 2 μM of the indicated protein for 1 h before the cellular workup and measurements, as described previously. Flow cytometry values are provided as median fluorescence intensity for the lissamine rhodamine B channel; *n* = 20 000 total per condition with two biological replicates each (mean ± SEM). FCS values are provided in nM; *n* > 25 for each FCS condition with two biological replicates each (mean ± SEM). Statistical significance was assessed using the Brown–Forsythe and Welch one-way analysis of variance (ANOVA) followed by an unpaired *t* test with Welch’s correction. ****p ≤ 0.0001, ***p ≤ 0.001, **p ≤ 0.01, *p ≤ 0.05.

Membrane translocation machines that transit unfolded protein domains sometimes tolerate secondary structures or even folded proteins if they are small and compact.^18,39,40^ Moreover, proteins with high pI’s (excess cationic surface charge) can engage negatively charged phospholipids for enhanced cellular uptake.^41,42^ Small stable protein domains, whether natural, evolved, or designed, are desirable research tools and are increasingly represented in clinical trials.^43^ Indeed, ZF5.3 was recently shown to facilitate cytosolic delivery of a nanobody-derived PROTAC that catalytically induces the degradation of BCL11A and upregulates fetal hemoglobin production, although the delivery efficiency was not evaluated.^9^ To more quantitatively evaluate whether small, stable proteins could be delivered effectively by ZF5.3, we turned to synthetic proteins derived from the fibronectin type III domain (monobodies). Monobodies can be engineered to display exceptionally high affinity for difficult-to-inhibit proteins,^44,45^ are 20–25% more compact than nanobodies,^45^ and are not themselves cell-permeant.^46,47^ In particular, we focused on NS1 (Figure 4a), a small (12 kDa), cationic (pI = 9.2 when conjugated to ZF5.3) monobody that binds HRAS and KRAS with high affinity (*K*_D_ values of 15 and 65 nM, respectively) and inhibits KRAS-driven tumor growth when expressed in vivo.^47^

NS1 and NS1–ZF5.3 were expressed, purified, and labeled at the C-terminus with rhodamine via a thiol-Michael addition reaction (Figures S8a and S8b), and characterized by LC/MS and CD (Figures S8c and S8d). Comparison of the wavelength spectra for NS1 and NS1–ZF5.3 suggests that the addition of ZF5.3 does not significantly perturb the secondary structure of NS1. As expected, both NS1 and NS1–ZF5.3 are highly thermostable (Figure 4a; *T_m_ = 73.2 °C for NS1 and 72.6 °C for NS1–ZF5.3). To evaluate delivery, Saos-2 cells were treated with 1–2 μM NS1^Rho^ and NS1–ZF5.3^Rho^ for 1 h, washed and trypsinized, and analyzed by flow cytometry and FCS (Figure 4b). Both the total uptake of NS1–ZF5.3^Rho^ and its ability to reach the cytosol were substantially higher than that of NS1^Rho^ (Figure 4b). The total uptake of NS1 was improved by 63–117-fold upon conjugation to ZF5.3, whereas delivery to the cytosol was improved by 7–12-fold. NS1–ZF5.3^Rho^ reached maximal cytosolic concentrations of 122.9 and 268.3 nM with starting incubation concentrations of 1 and 2 μM, respectively, yielding a delivery efficiency of 12.3–13.4%. Under equivalent conditions, this cytosolic concentration is roughly equal to that of the midstable SNAP variant ZF5.3–AGT54^Rho^ (Figure 3d), which has a significantly lower *T_m_ (46 °C) but also a less cationic pI (8.8) and a higher molecular weight (23.6 kDa). Given that the total uptake is significantly higher for NS1–ZF5.3 than ZF5.3–AGT54, these results suggest that a cationic surface charge and compact fold can result in modest cytosolic delivery, but the specific step(s) at which ZF5.3 conjugates escape the endocytic pathway is most efficient for easily unfoldable proteins.

### HOPS Provides a Portal for Efficient Endosomal Escape of Easily Unfolded Proteins

**Figure 5:**
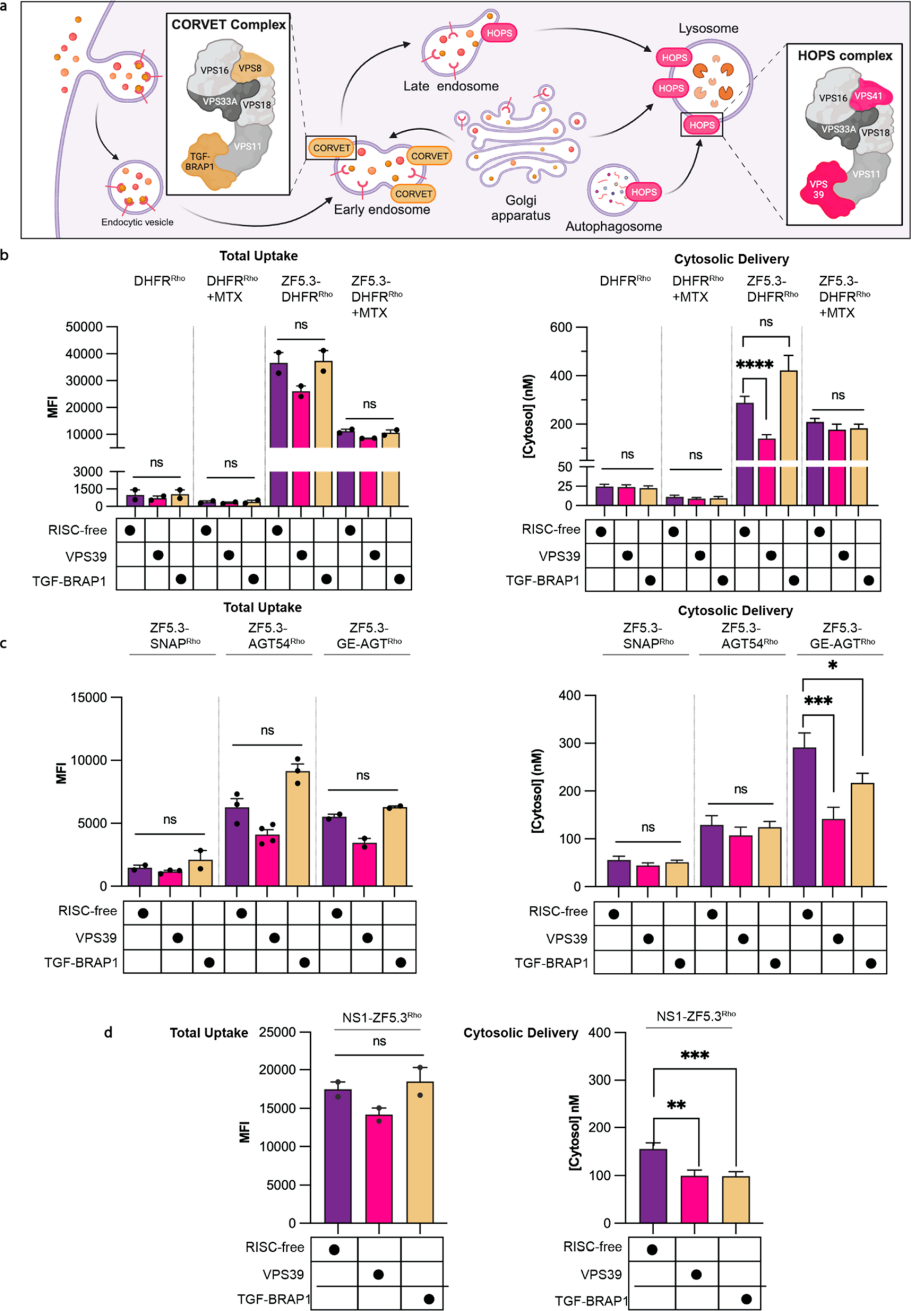
Easily unfolded proteins require HOPS and ZF5.3 to reach the cytosol efficiently. (A) Knockdowns were performed for the VPS39 subunit of the HOPS complex or the TGF-BRAP1 subunit of the CORVET complex. Both complexes participate in membrane tethering for either Rab5+ early endosomes and maturing endosomes (CORVET) or Rab7+ and Lamp1+ late endosomes and lysosomes (HOPS). Schematic adapted from “Role of HOPS in Lysosome Formation”, by BioRender.com (2023). (B–D) Plots illustrating the effects of VPS39 and TGF-BRAP1 knockdowns on total uptake (flow cytometry, median fluorescence intensity) and cytosolic access (FCS, nM) for DHFR proteins (B), SNAP-tag variants (C), and NS1–ZF5.3 (D) relative to a RISC-free negative control. Two biological replicates were performed for each experiment; *n* = 20 000 per condition in total for flow cytometry and *n* > 15 per condition for FCS. Error bars represent the SEM (****P < 0.0001, ***P < 0.001, **P < 0.01, *P < 0.05, and not significant (ns) for *P* > 0.05) from one-way ANOVA with unpaired *t* test with Welch’s correction.

Given the evidence that efficient ZF5.3-mediated membrane translocation demands protein unfolding, we next asked whether this delivery pathway makes use of endosomal machinery. We were specifically interested in the role of the HOPS and CORVET complexes, two essential hexameric tethering complexes involved in endosomal maturation events.^50,51^ HOPS coordinates with SNARE proteins and a Rab GTPase to drive late endosome–lysosome fusion, while CORVET performs an analogous role for early endosomal fusion (Figure 5a).^10,52,53^ Previous work revealed that efficient endosomal escape of ZF5.3, both alone and when fused to the intrinsically disordered cargo MeCP2, requires HOPS but not CORVET, suggesting an escape portal is generated during or after endolysosomal fusion.^5,6^ Whether this dependency extended to all ZF5.3 cargoes or only those that easily unfolded remained unclear.

We began by investigating the HOPS dependence of ZF5.3-mediated delivery of DHFR in the presence and absence of MTX. Saos-2 cells were transfected with siRNAs targeting either an essential HOPS subunit (VPS39) or the analogous CORVET subunit (TGF-BRAP1), as well as a nontargeting siRNA (RISC-free) as a negative control. All knockdowns were verified using qPCR (Figure S9). We then treated cells with 500 nM DHFR^Rho^ or ZF5.3–DHFR^Rho^ for 1 h and analyzed each sample by flow cytometry and FCS (Figure 5b). Although depletion of VPS39 had only a modest effect on the total uptake of either DHFR^Rho^ or ZF5.3–DHFR^Rho^, it substantially (51%) decreased the efficiency with which ZF5.3–DHFR^Rho^ trafficked to the cytosol relative to the RISC-free control (Figure 5b). Interestingly, knockdown of TGF-BRAP1 slightly increased the fraction of ZF5.3–DHFR^Rho^ that reached the cytosol (Figure 5b), a pattern also observed for ZF5.3^Rho^ alone^5^ but not for ZF5.3–MeCP2.^6^ Notably, VPS39 knockdown had no effect on the cytosolic delivery of ZF5.3–DHFR^Rho^ in the presence of one equivalent of MTX, nor any effect on the delivery of DHFR^Rho^. These results demonstrate that ZF5.3–DHFR, like ZF5.3 alone and ZF5.3–MeCP2, makes use of an intrinsic HOPS activity to reach the cytosol. The lack of HOPS dependence for ZF5.3–DHFR^Rho^ in the presence of MTX, as well as DHFR^Rho^ (± MTX), suggests that certain proteins escape endosomes inefficiently through one or more pathways but that attachment of ZF5.3 to a protein that easily unfolds biases endosomal escape toward a highly efficient, HOPS-dependent route.

To establish whether the link between HOPS and protein unfolding could be applied to other proteins, we examined the effect of HOPS- and CORVET-specific siRNA depletions on the uptake and cytosolic trafficking of SNAP-tag variants (Figure 5c). As observed for DHFR^Rho^ and ZF5.3–DHFR^Rho^, the depletion of VPS39 had no statistically significant effect on the uptake of any SNAP variant. Depletion of VPS39 also had no effect on the cytosolic delivery of the high-*T_m_ and mid-*T_m_ SNAP variants (ZF5.3–SNAP^Rho^ and ZF5.3–AGT54^Rho^); in all cases, the concentration established in the cytosol was relatively low (44–56 nM for ZF5.3–SNAP^Rho^ and 107–130 nM for ZF5.3–AGT54^Rho^). Depletion of VPS39 did, however, significantly decrease the level of cytosolic trafficking of the low-*T_m_ SNAP variant (ZF5.3–GE-AGT^Rho^) by 51.4%. Knockdown of TGF-BRAP1 had no effect on the delivery of the high- and mid-*T_m_ variants and a mild but statistically significant decrease (25.5%) in the delivery of the low-*T_m_ variant. The untagged SNAP^Rho^ variants reached extremely low cytosolic concentrations under all conditions tested and were too low to reliably quantify. For consistency, we also evaluated the effect of VPS39 and TGF-BRAP1 knockdown on NS1–ZF5.3^Rho^ delivery (Figure 5c). Depletion of both VPS39 and TGF-BRAP1 had a minimal effect on total uptake and a modest and statistically significant (36% and 37%, respectively) reduction in cytosolic concentration of NS1–ZF5.3^Rho^. The variable effect of TGF-BRAP1 knockdown on the delivery of ZF5.3–DHFR^Rho^, ZF5.3–GE-AGT^Rho^, and NS1–ZF5.3^Rho^ likely indicates some complexity in how the endosomal maturation machinery is utilized. Together, these data suggest that ZF5.3 conjugates with easily unfolded cargo exploit a high-efficiency, HOPS-dependent pathway that can be partially adopted by cargoes with high thermal stabilities provided the folded state is sufficiently compact and cationic. Even in this case, however, the delivery efficiency is markedly lower than that of a protein that can unfold under physiological conditions (see Discussion).

### STED Microscopy Reveals Membrane-Associated Subcompartments within Endolysosomes

But how does HOPS, which catalyzes homotypic and heterotypic membrane fusion from the cytosol, communicate, directly or indirectly, with material located within the endosomal lumen? Two lines of evidence suggest that endosomal escape involves more than the establishment of a membrane defect during vesicle fusion. First, efficient endosomal escape demands a covalent link between ZF5.3 and the delivered cargo.^8^ Second, ZF5.3 does not promote endosomal escape of other endosomally sequestered material.^5^ Although both ZF5.3^5^ and ZF5.3–DHFR localize primarily within the lumen of Lamp1+ endolysosomes when evaluated using confocal microscopy (Figure S10a), TauSTED microscopy of ZF5.3–DHFR^Rho^ treated cells (Figure S10b) revealed fluorescent populations that resemble intraluminal vesicles (ILVs, Figure S10c). Notably, at super-resolution the fluorescent subpopulations all appear near endolysosomal membranes (Figure S10c), suggestive of membrane interactions that facilitate endosomal release along a concentration gradient into the cytosol. Precisely how luminal ZF5.3–DHFR communicates with HOPS to ultimately cross the endosomal membrane and whether this mechanism proceeds via membrane interactions with ZF5.3 are areas of active investigation.

## Discussion

Here we describe the first design rules for efficient endosomal escape of cargo proteins conjugated to the cell-permeant miniature protein ZF5.3. We find that the efficiency of ZF5.3-mediated protein delivery to the cytosol is highest when the protein cargo readily unfolds under physiological conditions. Similar findings that low thermodynamic stability enhances intracellular delivery have been reported for toxin-mediated delivery of DARPins^54^ and even cytosolic penetration of antisense oligonucleotides,^55^ suggesting that the relationship between folding and endosomal escape may apply broadly to the passage of therapeutic macromolecules across cellular membranes.

**Figure 6:**
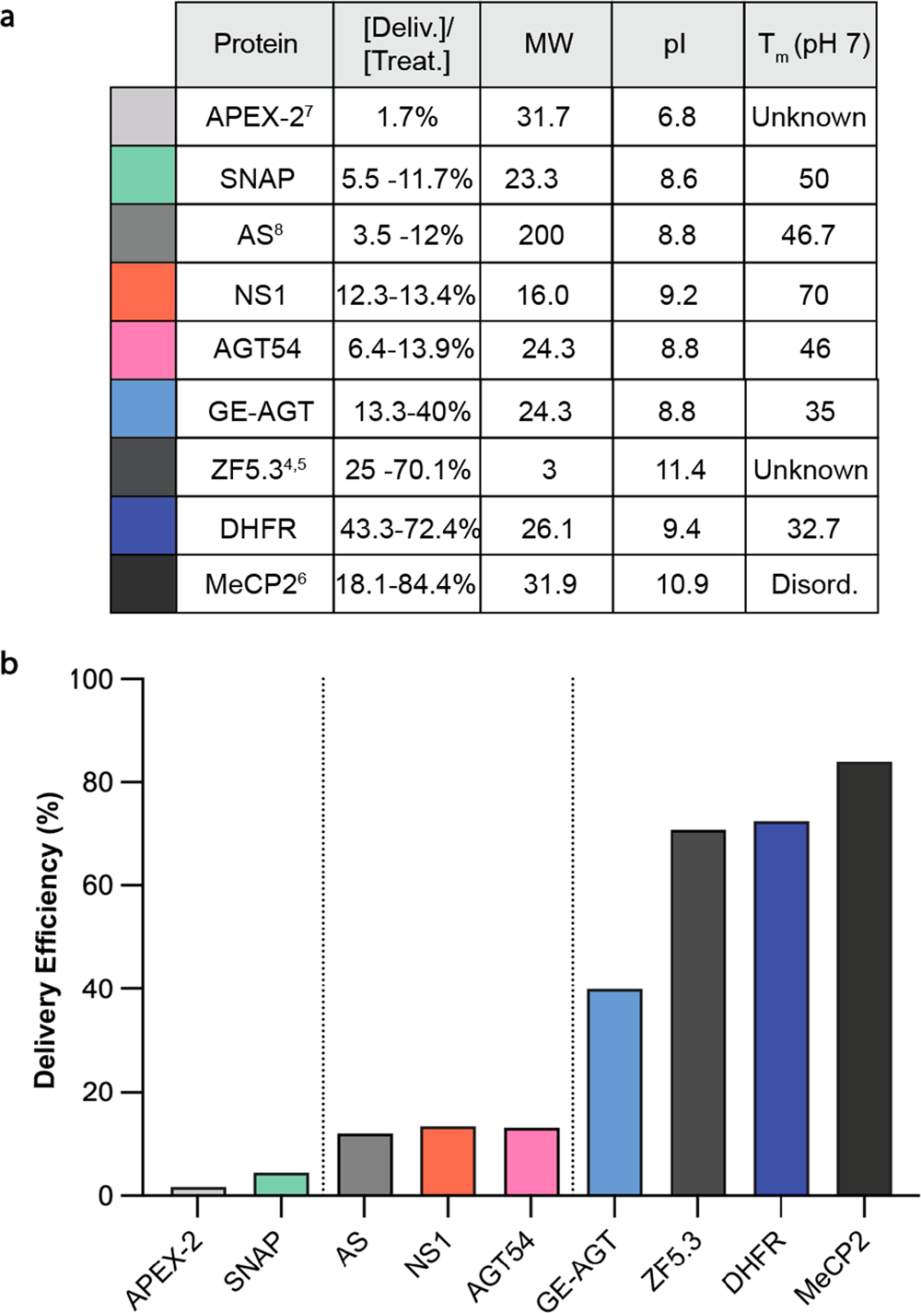
Efficient delivery of covalent ZF5.3 conjugates correlates directly with melting temperature. (A) Biophysical parameters and delivery efficiency for ZF5.3 alone (dark gray) or when conjugated to protein cargoes. Delivery efficiency is defined as the concentration that reaches the cytosol (or nucleus, for MeCP2) divided by the treatment concentration; the range is reported for all conditions tested. Molecular weight (MW) is defined in kilodaltons, pI is the isoelectric point, and *T_m_ is the apparent melting temperature, determined experimentally when conjugated to ZF5.3. (B) Graphical representation of the maximal delivery efficiency for ZF5.3-tagged cargoes listed in (a). Proteins with high *T_m_ values that are larger than 20 kDa are delivered with the lowest efficiency. Proteins with a *T_m_ ≈ 46 °C, or a high *T_m_ but small molecular weight, are delivered with midrange efficiency. Only proteins with a *T_m_ < 35 °C or that are intrinsically disordered are delivered with the highest efficiency.

Other groups have demonstrated that additional biophysical features, such as surface charge (measured by isoelectric point, pI) and molecular weight (MW), influence intracellular protein delivery.^41,42,56^ Our results strongly suggest that thermal stability is the most significant predictor of efficient endosomal escape, especially through a HOPS-dependent portal. This point is highlighted first by the observation that two ZF5.3–protein conjugates with equal thermal stabilities but very different molecular weights, ZF5.3–AS (200 kDa) and ZF5.3–AGT54 (24 kDa), are delivered with equal efficiencies (defined as the concentration established in the cytosol divided by the treatment concentration, Figure 6). Conversely, two ZF5.3–protein conjugates with equal molecular weights but different thermal stabilities, ZF5.3–SNAP (*T*_m_ = 50 °C) and ZF5.3–GE-AGT (*T*_m_ = 35 °C), are not delivered equally; the low-*T*_m_ protein is delivered efficiently (40%) whereas the high-*T*_m_ protein is not delivered (11%) (Figure 6).

We find that a low MW and high cationic charge can partially compensate for an unfavorably high *T*_m_ to improve cytosolic localization, as observed for NS1–ZF5.3; however, these attributes are insufficient to drive efficient delivery to the levels seen with intrinsically disordered or low-*T*_m_ cargo proteins. A close examination of our studies using NS1–ZF5.3 reveals two key patterns that further suggest delivery depends more on thermal stability than size or surface charge (Figure 6). First, although the cytosolic concentrations established by NS1–ZF5.3 (*T_m_ = 73 °C) and ZF5.3–AGT54 (*T_m_ = 46 °C) are similar, the overall uptake, as measured by flow cytometry, is different. The uptake of NS1–ZF5.3 is high, presumably because it possesses a higher pI, whereas the uptake of ZF5.3–AGT54 is relatively low. Thus, although NS1–ZF5.3 and ZF5.3–AGT54 reach the cytosol equivalently, the degree of endosomal escape is much higher for low-*T*_m_ ZF5.3–AGT54. Second, when comparing NS1–ZF5.3 (*T_m_ = 73 °C) with ZF5.3–DHFR (*T_m_ = 32 °C), the total amount of endocytosed protein is nearly equal (indeed, the pI values of both proteins are almost identical), but the fraction of ZF5.3–DHFR that reaches the cytosol is >3-fold higher. These comparisons suggest that cationic charge may stimulate overall uptake but the efficiency of endosomal escape is highest for low-*T*_m_ proteins regardless of size or charge, at least when conjugated to ZF5.3.

There are dozens of annotated proteins with *T*_m_ values comparable to those chosen in this study^57^ and hundreds of proteins containing >40% intrinsic disorder.^58^ Protein engineering efforts to introduce pH- or temperature-dependent destabilizing mutations into otherwise ideal therapeutic candidates to improve ZF5.3-mediated delivery, such as NS1, may be a viable strategy to enhance the delivery efficiency. The observation that ZF5.3-mediated endosomal escape is most efficient when conjugated to low-*T*_m_ proteins, and that this pathway demands communication between luminal ZF5.3 and cytosol-facing HOPS, suggests the existence of a selective portal through which membrane transport occurs. In nearly all cases, nature mediates such transport via a proteinaceous channel embedded within the membrane, such as the recently reported perforin-2 channel in dendritic cells.^59^ Whether ZF5.3 accomplishes its escape via lipid interactions or makes use of a yet-undetected protein channel remains under active investigation. Regardless, the results of this study provide clear biophysical guidelines to promote endosomal escape of ZF5.3-tagged cargo and can be applied to the development and expansion of novel protein therapies.

## Supporting Information Available

Reagents and chemicals, detailed methods for all experimental procedures, Tables S1–S3, Figures S1–S10, and references (PDF)
